# Risk stratification by 30-day prognostic factors of clinical outcomes after granulocyte transfusion in acute myeloid leukemia: A single-center retrospective study

**DOI:** 10.1371/journal.pone.0273827

**Published:** 2022-08-30

**Authors:** Jaeeun Yoo, Hyung Suk Cho, Jae-Ho Yoon, Byung Sik Cho, Hee-Je Kim, Dong-Gun Lee, Dong Wook Jekarl, Myungshin Kim, Eun-Jee Oh, Yeon-Joon Park, Yonggoo Kim

**Affiliations:** 1 Department of Laboratory Medicine, Incheon St. Mary’s Hospital, College of Medicine, The Catholic University of Korea, Seoul, Korea; 2 Department of Laboratory Medicine, Seoul St. Mary’s Hospital, College of Medicine, The Catholic University of Korea, Seoul, Korea; 3 Department of Laboratory Medicine, Apheresis Unit, Seoul St. Mary’s Hospital, College of Medicine, The Catholic University of Korea, Seoul, Korea; 4 Department of Internal Medicine, Catholic Hematology Hospital, Seoul St. Mary’s Hospital, College of Medicine, The Catholic University of Korea, Seoul, Korea; 5 Division of Infectious Diseases, Department of Internal Medicine, Seoul St. Mary’s Hospital, College of Medicine, The Catholic University of Korea, Seoul, Korea; 6 Research and Development Institute for In Vitro Diagnostic Medical Devices, College of Medicine, The Catholic University of Korea, Seoul, Korea; Istanbul University-Cerrahpaşa, Cerrahpaşa Faculty of Medicine, TURKEY

## Abstract

**Background:**

Granulocyte transfusions (GTs) have been used to treat infections in neutropenic patients undergoing chemotherapy or hematopoietic stem cell transplantation. However, there is persistent controversy regarding their outcomes. We aimed to analyze accumulated clinical and laboratory data from patients with acute myeloid leukemia (AML) who underwent GT at our institution in the last 10 years to determine optimal parameters to estimate the GT effect. We hypothesized that patients grouped according to prognostic factors would have inconsistent clinical outcomes.

**Materials and methods:**

In this single-center retrospective study, we collected medical records of 219 GT-treated patients diagnosed with AML from 2009 to 2019. Prognostic factors, including clinical and laboratory parameters, were assessed. Serial measurements of laboratory parameters before and after GT were collected, and the area under the curve of the white blood cells (AUC-WBC) was calculated using the trapezoidal method. A prognostic scoring system using 8 factors from multivariate analysis was analyzed. The primary outcome was survival at 30 days (D30) after GT initiation.

**Results:**

The 8 factors for the prognosis scoring system included secondary AML, mean AUC-WBC, prothrombin time, and levels of blood urea nitrogen (BUN), bilirubin, alanine aminotransferase (ALT), phosphorus, and lactate dehydrogenase (LDH). Patients were grouped into 4 risk groups (low, medium, high, and very high), and the D30 survival rates for each group were as follows: 87.6% (99/113), 55.9% (33/59), 21.1% (4/19), and 0% (0/19), respectively. Hematopoiesis, liver, and renal function affected the outcome. *FLT3* mutation acted as a favorable factor for D30 survival.

**Conclusions:**

GT response in patients with AML seemed to be reflected by 8 score markers, and GT was significantly effective in the low-risk group. We suggest that it is important to evaluate the risk assessment of patients before GT to achieve better outcomes.

## Introduction

Prolonged neutropenia results from myelosuppressive chemotherapy or stem cell transplantation in patients with hematological malignancies. Neutropenia leads to vulnerability to life-threatening bacterial and fungal infections [1, 2]. The use of antimicrobial therapeutics remains the primary treatment strategy for these infections. Granulocyte transfusion (GT) can be an adjunctive therapeutic method when patients are unresponsive to these therapeutic modalities. The use of GT to replenish cells until the recovery of hematopoiesis in bone marrow is an appealing treatment modality [3, 4].

Although GT has a long history, the definite benefit of GTs has not been shown or is inconclusive. Many physicians were unconvinced that GT showed meaningful clinical improvement in their patients. The heterogeneity of donors or recipients in GT, types of infection, antimicrobial therapy, dosage of GT, and other variations might have hindered modern randomized controlled trials [5–8]. In addition, GT has been prescribed inconsistently in patients for whom there is no effective antimicrobial therapy.

White blood cells (WBCs), mostly neutrophils, have a lifespan of 5 days. Among them, 95% of the cells are in the bone marrow, 3% are in the marginal pool of peripheral blood vessels, 2% are circulating, and 6.0 × 10^10^ cells are replaced daily [1, 9]. Neutrophils play a key role in the first line of defense against bacteria and fungi. Neutrophils eradicate microbes by phagocytosis and formation of neutrophil extracellular traps [9–11]. Neutrophils express or produce approximately 20 chemokines, 21 cytokines, and 30 other molecules [11–13].

As neutrophils secrete soluble molecules related to immune modulatory functions, we hypothesized that WBCs could be viewed as pharmaceutical agents. This view emphasizes the functional secretory aspect of WBCs. Assessment of these functional aspects of WBCs can be analyzed using the area under the curve (AUC). In pharmacokinetics, the AUC is defined as the drug concentration across time that is integrated. The AUC can represent drug exposure over time, followed by the biological effect of a drug [14, 15].

The AUC of WBCs (AUC-WBC) was calculated by integrating the serial WBC count across the measured time point during infection. AUC-WBC seemed to reflect the immunologic state of the patients compared to the WBC count. Indeed, these AUC-WBC values were evaluated in patients with aplastic anemia treated with GT [16]. However, the effectiveness of GT was inconclusive owing to the small size of the study population (n = 32) or the possible ineffectiveness of GT.

In this study, we analyzed accumulated clinical and laboratory data from patients with acute myeloid leukemia (AML) who underwent GT at our institution in the last 10 years to determine optimal parameters to estimate the GT effect. We hypothesized that patients grouped according to prognostic factors would have inconsistent clinical outcomes.

## Materials and method

### Ethics statements

This single-center, retrospective study was approved by the Institutional Review Board (IRB) of Seoul St. Mary’s Hospital (IRB number: KC20WISI0178). The need for informed consent was waived by the IRB because of the retrospective nature of the study.

### Patients

We searched electronic medical records between April 2009 and December 2019. The data of 11,457 patients diagnosed with hematologic diseases were retrieved using a centralized electronic data repository at our institution, namely, the clinical data warehouse (S1 Fig in S1 File). Among them, 282 patients who underwent GTs at our institution were retrospectively identified, and 219 patients with AML were eligible for inclusion in this study. A diagnosis of AML before 2016 was reclassified according to the 2016 World Health Organization classification [17]. The patients were treated with the standard chemotherapy protocol suggested in previous reports, and microbial prophylaxis was administered to the candidate patients [18–20].

### Granulocyte transfusion protocol

At our institution, the GT protocol was as follows: granulocytes were collected from healthy voluntary donors after informed consent was obtained. To mobilize granulocytes in the donors, 10 μg/kg of granulocyte colony stimulating factor was injected subcutaneously approximately 12 hours before granulocyte collection. Acetaminophen (dose, 650 mg) was administered orally in the presence of symptoms, such as fever, headache, and chills. Granulocytes were collected using mononuclear cell collection protocols (LRS Turbo, version 7.0) and a COBE Spectra system (Terumo BCT, Lakewood, USA). Anticoagulant citrate dextrose solution A was mixed with blood at a ratio of 1:15. The collection speed was 30–45 mL/min, and the total volume of blood collected in a single collection was 5,500–7,000 mL. The hematocrit in the color gram was set to 5.0%, and the total volume was 350 mL [21]. The collected granulocytes were irradiated with gamma rays (25 Gy) from a cesium source and maintained at room temperature. Granulocytes were administered to patients when they met the following conditions: 1) proven or highly probable bacterial or fungal infection, 2) no response to appropriate antimicrobial therapy, defined as failure to reach neutrophil recovery (>1.0 × 10^9^/L) within 72 hours, and 3) absolute neutropenia (<0.5 × 10^9^/L) [7, 19]. The GT procedures were continued until clinical improvement (e.g., resolution of fever (<38°C), conversion of cultures to a negative result, and healing of involved sites), which was determined by the physicians.

### Clinical and laboratory data collection

To determine the optimal parameters for GT, clinical data of patients with AML and laboratory data before and after GT were retrieved from every GT session. Laboratory data of complete blood counts, coagulation, and blood chemistry and microbiological data were collected.

Transfusion data were collected for the platelet concentrate, apheresis platelets, and packed red blood cells (RBCs) during the admission period for GT. The platelet unit was counted as an apheresis platelet unit, and the platelet concentrate was regarded as 1/6 of the unit. RBC transfusion included filtered RBCs or non-filtered RBCs, and 350 mL or 400 mL of RBCs was regarded as 1 unit.

While collecting the data, we also calculated the AUC of the WBC count for each patient using serial WBC data. WBC counts before and after GT were plotted for survivors (S2 Fig in S1 File) and nonsurvivors (S3 Fig in S1 File). The functional aspect of WBCs injected with GT might be better represented using the AUC than the WBC count alone.

We divided the patients into 2 groups, survivors and nonsurvivors, at day 30 (D30) after GT and searched for any significant differences.

### Outcome measures

The primary outcome was D30 survival after GT. As secondary outcomes, variables associated with the clinical and laboratory parameters were studied.

### Survival analysis and prognosis scoring

The cut-off point for laboratory data was determined by receiver operating characteristic (ROC) curve analysis (S1 Table in S1 File) based on the D30 survival state. Using the maximum cut-off value from the ROC curve, patients were categorized into 2 groups. Kaplan–Meier analysis was performed to compare the data between the 2 groups, and survival curves were plotted (S2 Table and S4 Fig in S1 File).

Next, parameters with statistical significance from survival analysis were collected and used as variables for D30 survival using univariate and multivariate Cox proportional hazard regression models. Finally, we established a scoring system according to the hazard ratio (HR) values of the independent variables (S3 Table in S1 File). The risk score was assigned according to the beta coefficient by regression analysis.

### Statistical analysis

R software, version 4.0.1 (R Foundation, Vienna, Austria) was used for data handling and processing. ROC curve analysis was performed using the pROC R package [22], and the cut-off lines were used for Kaplan–Meier analysis and compared with the log-rank test using the survival and survminer R packages [23, 24]. The ggplot R package was used for figure plotting [25]. Serial data before GT were averaged to represent the patients. For statistical analysis of the mean or median, the Student’s t-test and Mann–Whitney U test were performed to analyze continuous variables with a normal distribution and those with a non-normal distribution, respectively. For categorical variables, the chi-square test was used, and if >20% of the cells had a frequency <5, the Fisher’s exact test was used. For the association between variables and AUC-WBC, univariate analysis followed by multivariate analysis was performed using linear regression analysis. For prognostic factor analysis, univariate analysis using Cox regression analysis for each variable was performed, followed by multivariate analysis to investigate the association between the parameters and D30 survival of the patients. The HR and corresponding 95% confidence interval were computed. All analyses were performed using SPSS, version 24 (IBM Corp., Armonk, NY, USA).

The AUC was calculated using the trapezoidal method as follows: trapezoidal area = [(C_i+1_ + C_i_) / 2] × (T i+1 –T_i_) and AUC-WBC = $\sum_{i}^{n}C_{i}T_{i}$, where C indicates the WBC count and T indicates the time of the WBC count measurement. AUC-WBC was divided by the number of transfused granulocyte units, which are denoted as the mean AUC-WBC [14, 15]. All tests were two-sided, and *P*-values <0.05 were considered statistically significant.

## Results

### Differences in clinical and laboratory finding between D30 survivors and nonsurvivors

The clinical differences between the 2 groups divided according to survival at D30 from the GT were statistically evaluated (Table 1). AML with myelodysplasia-related changes (53.1%, 17/32) and secondary AML (59.1%, 13/22) showed a significantly higher frequency in D30 nonsurvivors. Among the patients with secondary AML, myelodysplastic syndrome was the most common preceding hematologic malignancy (46.1%, 12/22) (S4 Table in S1 File).

**Table 1:** Clinical differences between survivors and nonsurvivors at day 30 after granulocyte transfusion.

	N	Total	D30 survivor	D 30 nonsurvivor	*P* value
			(n = 137)	(n = 82)	
Age (year)	219	47.5 ± 13.8	46.7 ± 13.1	48.9 ± 14.8	NS
Female / Male, n (%)	219	79 / 140	47 / 90	32 / 50	NS
AML subtype	219				
AML with *CBFB/MYH11*	8	8	4	4	NS
AML with *PML/RARA*^a^	8	8	4	3	NS
AML with *RUNX1/RUNX1T1*	22	22	16	6	NS
AML with *KMT2A* abnormalities	7	7	6	1	NS
AML with *CEBPA* mutation^b^	4	4	2	2	NS
AML with *NPM1* mutation^b^	14	14	9	5	NS
AML, MRC	32	32	15	17	0.047
AML, NOS	102	102	64	38	NS
Secondary AML	22	22	9	13	0.031
Cytogenetic abnormalities	219				NS
0 / 1		84 / 71	55 / 41	29 / 30	
2 / 3		30 / 34	20 / 21	10 / 13	
Genetic study					
*BAALC* level	189	8.3 ± 30.7	10.5 ± 38.2	4.6 ± 8.2	NS
*WT1* level	193	0.97 ± 2.45	1.03 ± 2.92	0.88 ± 1.39	NS
*cKIT* mutation (no / yes)	121	91 / 30	55 / 19	36 / 11	NS
*FLT3 ITD* mutation (no / yes)	171	142 / 29	83 / 20	59 / 9	NS
Induction CTX					
antracyclin, cytarabine / others	211	199 / 12	126 / 7	73 / 5	NS
Reinduction CTX					
FLANG / others	74	26 / 48	19 /32	7 / 16	NS
Disease state at GTX	219				NS
Newly diagnosed		171	115	56	
Relapse		48	28	20	
Microbial parameters					
Microbe growth (no / yes)	219	58 / 161	43 / 95	15 / 66	NS
Growth frequency (0 / 1 / 2)	219	55 / 67 / 97	40 / 45 / 52	15 / 22 / 45	0.042
Recovered microbe	291	291	167	124	NS
Gram-positive / Gram-negative		179 / 102	107 / 55	72 / 47	
fungal, yeast		10	5	5	NS
Probable fungal infection ^c^		13	12	1	NS
Microbe isolation site					
Blood (no / yes)	200	110 / 90	70 / 55	40 / 35	NS
Lung (no / yes)	200	182 / 18	113 / 12	69 / 6	NS
Transfusion parameters					
AN to GT (d)	186	25.3 ± 12.6	26.5 ± 11.7	23.9 ± 13.6	NS
Proven culture to GT (d)	186	12.6 ± 13.3	14.1 ± 15.3	11.3 ± 11.0	NS
GT (unit, 1 / 2 / 3 / ≥4)	219	41 / 27 / 17 / 134	20 / 18 / 12 / 87	21 / 9 / 5 / 47	NS
RBC transfusion (unit)^d^	171	12.4 ± 10.5	12.0 ± 13.1	13.1 ± 13.1	NS
PLT transfusion (SDP unit)^e^	171	20.9 ± 19.8	20.5 ± 17.9	21.4 ± 22.7	NS

^a^One case of AML with *ZBTB16/RARA* was included.

^b^*CEBPA* and *NPM1* gene sequencings were performed for 53 and 135 of the 219 patients, respectively. The biallelic mutation of *CEBPA* gene is denoted.

^c^Probable fungal infections are diagnosed by radiologic findings mostly on computed tomography.

^d^Transfusion of RBCs included filtered or non–filtered RBCs. One unit of RBC was 320 mL or 400 mL. The total transfusion amount was calculated for newly diagnosed patients during their first admission, before or after GT.

^e^A unit of platelets was based on a single donor. The platelet concentration was divided by 6 before the calculation. The total transfusion amount was calculated for newly diagnosed patients during their first admission, before or after GT.

NS, not significant; AML, acute myeloid leukemia; MRC, myelodysplasia–related changes; AN, absolute neutropenia; CTX, chemotherapy; d, day; GT, granulocyte transfusion; SDP, single–donor platelet; FLANG, fludarabine, cytosine arabinoside, mitoxantrone, granulocyte colony stimulating factor.

Increased frequency of microbial growth was also associated with higher D30 mortality. Statistical analysis of the other clinical parameters showed no significant difference between the groups. GT after neutropenia, duration from microbial growth, and GT were not associated with D30 mortality. The amount of GT was not different between D30 survivors and nonsurvivors. Among the 219 patients, *Enterococcus faecium* (31.6%, 45/142) was the most common pathogen recovered from the blood culture. *E*. *coli* (26.5%, 17/64) was the most common pathogen recovered from catheter specimens, and *Acinetobacter baumannii* (17.4%, 4/23) and *Staphylococcus aureus* (17.4%, 4/23) were the most common pathogens recovered from respiratory specimens (S5 Table in S1 File).

In total, 2464 serial measurements from 219 patients with AML were retrieved. The mean AUC-WBC [interquartile range] values for D30 survivors and D30 nonsurvivors were 9.42 [2.54–19.61] and 5.73 [0.58–12.43], respectively, with statistical significance (*P* = 0.003). AUC-WBC and protein and albumin levels were lower in D30 nonsurvivors than in D30 survivors. Prothrombin time (PT)-activated partial thromboplastin time (aPTT), total bilirubin, direct bilirubin, aspartate aminotransferase (AST), alanine aminotransferase (ALT), phosphorus, sodium, and C-reactive protein (CRP) levels were increased in D30 nonsurvivors compared to D30 survivors (S6 Table in S1 File).

Laboratory data before and after GT in the 2 groups are plotted in (S5 Fig in S1 File). Most of the parameters showed statistical significance between survivors and nonsurvivors before GT. However, most of these laboratory parameters were similar and statistically insignificant after GT.

### Survival analysis and prognosis scoring

Survival analysis was performed to determine the prognostic parameters. The following parameters were statistically significant: secondary AML, WBC count, AUC-WBC, neutrophil count; lymphocyte count, hemoglobin level, hematocrit level, platelet count, red cell distribution width, PT, aPTT, creatinine level, protein level, albumin level, bilirubin level, direct bilirubin level, AST level, ALT level, lactate dehydrogenase (LDH) level, phosphorus level, chloride level, magnesium level, erythrocyte sedimentation rate (ESR), and CRP level. However, the frequency of microbial growth was not statistically significant.

**Fig 1:**
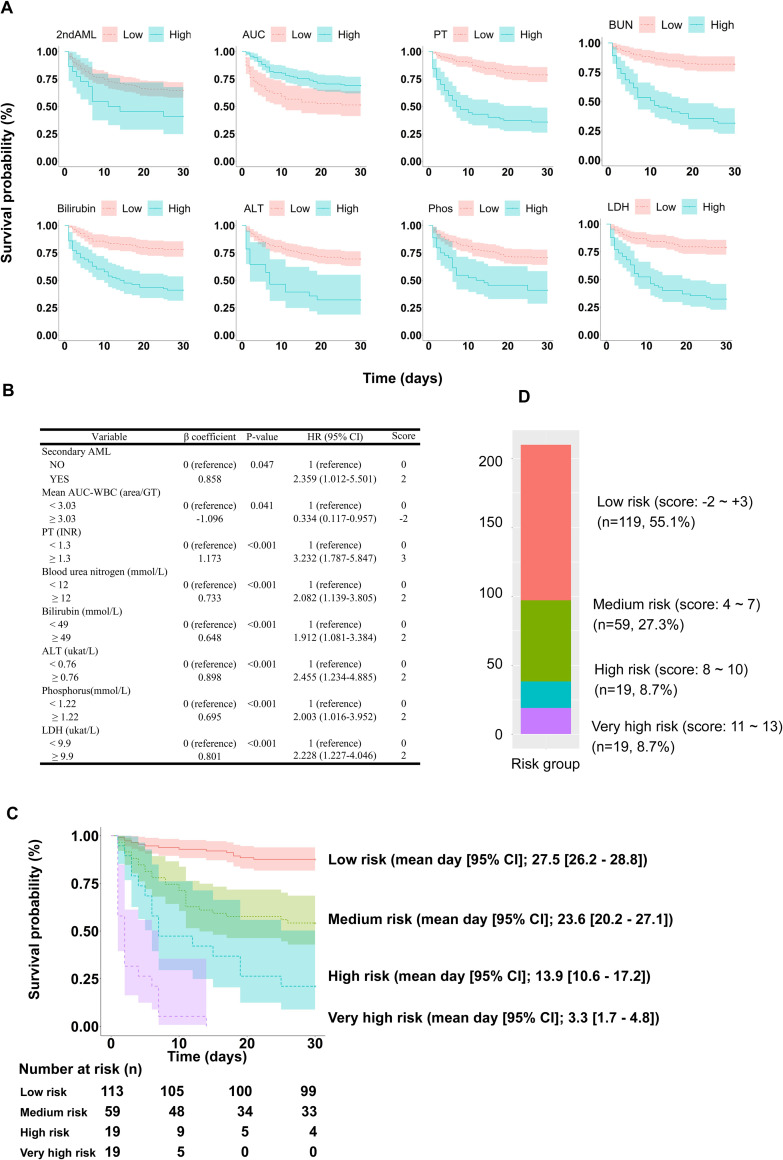
Prognosis scoring system for patients undergoing granulocyte transfusion. (A) Kaplan–Meier survival analysis of clinical and laboratory parameters are shown. (B) Eight factors after multivariate analysis for day 30 survival analysis. Scoring values correspond to the hazard ratio. (C) Survival analysis by the scoring system. (D) The 4 patient groups were low, medium, high, and very high–risk (n = 216). 2ndAML, secondary acute myeloid leukemia; AUC, area under the curve; PT, prothrombin time; BUN, blood urea nitrogen; ALT, alanine aminotransferase; Phos, phosphorus; LDH, lactate dehydrogenase; HR, hazard ratio.

Prognostic parameters with statistical significance were collected and used as variables for D30 mortality using univariate and multivariate Cox proportional hazard regression models. The findings are presented in Fig 1B. Eight parameters including secondary AML, AUC-WBC, BUN, ALT, bilirubin, PT, phosphorus, and LDH were associated with a significantly higher D30 mortality after GT (*P* < 0.05).

Based on multivariate analysis, we established a scoring system according to the HR values of the independent variables. The score was assigned according to the beta coefficient by regression analysis (Fig 1A and 1C). According to the scoring system, patients were divided into 4 risk groups (score): low (-2–3), medium (4–7), high (8–10), and very high (11–13). The Kaplan–Meier survival curves for each risk group are presented in Fig 1D, which shows that the survival rates were significantly different according to each group. The D30 survival rates for each group were as follows: low, 87.6% (99/113); medium, 55.9% (33/59); high, 21.1% (4/19); and very high, 0% (0/19).

The clinical characteristics and laboratory parameters of the 4 risk groups are shown in Table 2. We found that most of the laboratory parameters of the 4 groups showed statistically significant differences, but the clinical parameters were not significantly different. Only secondary AML, culture frequency, GT frequency, and RBC transfusions were significantly different between the groups. Laboratory parameters related to hematopoiesis and hepatic and renal functions seemed to affect the outcome.

**Table 2:** Clinical and laboratory parameters classified by the 4 risk groups.

	Low risk	Medium risk	High	Very High risk	*P*
	group	group	risk group	group	value
	(n = 113)	(n = 59)	(n = 19)	(n = 19)	
Age	49 [38–57]	59 [41–62]	52 [48–60]	47 [26–58]	
Sex, female	36 (31.9)	24 (40.7)	7 (36.8)	9 (47.4)	
Secondary AML	5 (4.8)	4 (7.7)	6 (33.3)	6 (35.3)	< 0.001
AML, MRC	12 (10.6)	8 (13.6)	6 (31.6)	3 (15.8)	
Relapsed AML	27 (26.2)	10 (19.2)	5 (27.8)	5 (29.4)	
Reinduction CTX	40 (35.4)	19 (32.2)	7 (36.8)	10 (52.6)	
*WT1* expression	0.18 [0.04–0.89]	0.39 [004–1.27]	0.38 [0.02–0.82]	0.18 [0.02–0.81]	
*BAALC* expression	2.04 [0.35–6.92]	1.44 [0.25–6.61]	0.49 [0.13–1.48]	0.88 [0.14–3.89]	
*FLT3* mutation	22 (25.0)	4 (8.2)	0 (0)	2 (13.3)	0.021
Karyotype					
Normal	43 (38.1)	22 (37.3)	8 (42.1)	9 (47.4)	
1–2 abnormality	56 (49.5)	25 (42.4)	10 (52.6)	8 (42.1)	
Complex	14 (12.4)	12 (20.3)	1 (5.3)	2 (10.5)	
Culture, blood	44 (42.7)	26 (48.1)	7 (41.2)	10 (55.6)	
Culture frequency ≥ 2	38 (33.6)	33 (55.9)	12 (63.2)	10 (52.9)	0.007
Gram (+) microbe	57 (55.3)	28 (51.9)	10 (55.6)	11 (61.1)	
GT	2 [4–7]	5 [2–9]	5 [2–8]	2 [1–4]	0.039
RBC Transfusion (U)	9 [5–12]	11 [7–18]	10 [6–14]	11 [7–18]	0.034
PLT Transfusion (U)	13 [7–21]	14 [8–35]	15 [10–29]	19 [10–29]	
Laboratory features					
AUC-WBC	9 [2.1–20.3]	6.8 [1.9–13.8]	4.9 [1.1–16.5]	1.7 [0.1–6.9]	0.009
mean AUC-WBC	1.8 [0.7–3.3]	1.3 [0.6–2.1]	1 [0.5–2.5]	0.4 [0.1–2.4]	0.004
WBC (10^9^/L)	1.71 [0.96–2.78]	1.39 [0.71–2.27]	0.68 [0.21–1.83]	0.67 [0.06–2.42]	0.015
RBC (10^12^/L)	3.37 [3.01–3.75]	3.25 [2.90–3.69]	3.22 [2.83–3.71]	2.87 [2.68–3.33]	0.017
Hg (g/L)	102 [91–114]	97 [86–110]	96 [85–112]	86 [80–99]	0.012
PLT (10^9^/L)	75 [40–122]	59 [26–92]	45 [28–82]	23 [6–60]	0.001
PT (INR)	1.18 [1.13–1.24]	1.33 [1.23–1.42]	1.39 [1.22–1.67]	1.52 [1.48–1.86]	< 0.001
aPTT (sec)	35.8 [31.9–39.9]	34.7 [31.9–42.3]	36.1 [33.0–48.1]	46.5 [40.4–51.8]	0.002
BUN (mmol/L)	6.85 [4.46–9.32]	12.25 [8.92–17.78]	51.9 [18.53–20.88]	19.21 [13.9–25.42]	< 0.001
Cr (umol/L)	55.69 [42.43–68.07]	69.84 [50.39–97.24]	93.7 [68.9–151.2]	1.55 [137.02–163.54]	< 0.001
protein (g/L)	57 [53–62]	56 [48–60]	52 [48–57]	49 [44–58]	< 0.001
Albumin (g/L)	29 [28–31]	27 [25–29]	28 [24–31]	26 [24–30]	< 0.001
AST (ukat/L)	0.33 [0.27–0.43]	0.45 [0.32–0.57]	0.95 [0.37–2.09]	0.83 0.42–6.96	< 0.001
ALT (ukat/L)	0.33 [0.25–0.43]	0.37 [0.28–0.57]	0.42 [0.25–1.00]	0.8 [0.32–1.90]	0.002
Bilirubin (umol/L)	18.8 [13.6–29.1]	65 [25.66–138.54]	88.9 [30.8–124.8]	109.4 [71.8–217.2]	< 0.001
uric acid (mmol/L)	0.11 [0.09–0.14]	0.14 [0.10–0.20]	0.18 [0.14–0.28]	0.22 [0.15–0.33]	< 0.001
Calcium (mmol/L)	1.98 [1.90–2.05]	1.98 [1.85–2.02]	1.95 [1.82–2.13]	2.02 [1.85–2.13]	
Phosphorus (mmol/L)	0.87 [0.74–1.07]	0.94 [0.81–1.16]	1.23 [1.10–1.49]	1.32 [1.13–1.58]	< 0.001
Sodium (mmol/L)	138 [136–141]	142 [138–149]	141 [137–143]	144 [138–149]	< 0.001
Potassium (mmol/L)	3.4 [3.1–3.7]	3.3 [3.1–3.8]	3.6 [3.4–3.9]	3.6 [3.1–4.2]	0.048
Chloride (mmol/L)	102 [99–104]	104 [101–109]	101 [98–104]	104 [101–109]	0.012
LDH (ukat/L)	5.83 [4.16–7.63]	8.53 [6.56–15.66]	15.92 [10.20–20.51]	13.71 [10.52–30.64]	< 0.001
Magnesium (mmol/L)	0.82 [0.74–0.86]	0.82 [0.78–0.91]	0.82 [0.78–0.91]	0.91 [0.74–0.91]	
CRP (mg/L)	153 [108–195]	192 [134–245]	193 [150–298]	187 [155–275]	< 0.001
ESR (mm/hr)	45 [22–60]	23 [14–41]	39 [16–51]	15 [3–47]	< 0.001

AML MRC, acute myeloid leukemia with myelodysplasia–related changes; CTX, chemotherapy; GT, granulocyte transfusion; RBC, red blood cell; PLT, platelet; AUC–WBC, area under the curve of white blood cells; Hb, hemoglobin; PT, prothrombin time; APTT, activated partial thromboplastin time; BUN, blood urea nitrogen; Cr, creatinine; LDH, lactate dehydrogenase; CRP, C–reactive protein; ESR, erythrocyte sedimentation rate.

## Discussion

In this study, we hypothesized that patients grouped according to prognostic factors would have inconsistent clinical outcomes. Eight parameters including secondary AML, mean AUC-WBC, BUN, ALT, bilirubin, PT, phosphorus, and LDH were associated with a significantly higher D30 mortality after GT. Also, patients grouped by prognostic factors showed significant D30 survival differences.

GT has been available in the clinical field for a long time; however, its clinical efficacy has not been decisively concluded. Many previous randomized controlled trials, including the Resolving Infection in Neutropenia with Granulocytes Study by Price et al. did not provide the definitive answer on the efficacy of GT [5]. GT had no overall effect on the primary outcome. However, enrollment was only half that planned; therefore, it is inappropriate to interpret the result that GT is ineffective.

RBC or platelet transfusion is focused on replenishing decreased cells, whereas GT is more complex. GT can be defined as a cellular therapy for infusing granulocytes, which can be represented by the cell count. As granulocytes play a central role in the immune system, GT not only replenishes granulocytes but also restores the impaired immune system or complex immune network. RBCs or platelets are functioning cells, whereas granulocytes construct the immune network by interrelating with other cells or eradicating microbes. The function of GT might take time, and other unknown variables might play a role, which requires further study.

The secretome of neutrophils is not fully understood, but the secretion of inflammatory mediators, granules, vesicles, and neutrophil extracellular traps contributes to immune function [26, 27]. The role of these mediators or the measurement of their function awaits elucidation. In this study, we assumed that the secretome or functional aspects of WBCs could be estimated using AUC-WBC. The functional aspect of neutrophils at the proteome level may enhance our understanding of the role of WBCs in the infectious state.

The WBC count might represent the phagocytic function of WBC or a function that was performed on a cell basis. However, AUC-WBC might represent the effect of the secretome of WBCs or WBCs as a collective measure of pharmaceutical agents. Indeed, AUC-WBC was a prognostic factor associated with D30 survival after GT in this study.

GT frequency was associated with the mean AUC-WBC but not with D30 survival. These data imply that GT is a prerequisite for maintaining the mean AUC-WBC. GT itself is only a sufficient condition that is not necessary for a favorable outcome [28–30]. AUC-WBC, not the WBC count or GT frequency, was associated with D30 prognosis, which might reflect that time is required for constructing an immune network or secretome for mitigating microbial burden and alleviating harmful effects of the inflammatory response.

Prognostic factors for D30 survival after GT included not only AUC-WBC but also secondary AML, BUN, ALT, bilirubin, PT, phosphorus, and LDH levels. The homogeneous group was clearly divided into 4 groups according to the prognosis scoring system, and their prognostic differences were evident. Our study findings might be attributed to the exact evaluation of the efficacy of complex GTs.

The prognosis scoring system clustered the patient group into 4 groups, resulting in low, medium, high, and very high-risk groups. The D30 survival rate in the very high-risk group was 0%, whereas that in the low-risk group was 87.6%. Evaluation of the efficacy of GT requires patient stratification based on prognosis. Prognostic scoring systems might support randomized controlled trials or further studies by adjusting patient and control groups with similar prognostic parameters.

It could be implied that various hematologic parameters and the transfusion of RBCs and granulocytes are associated with bone marrow function. Different bone marrow functions might be an underlying factor affecting the prognostic group. Immunologic function, bacterial clearance, and minimization of the harmful effects of inflammation also depict the prognosis of the GT group. This is contrary to the intuition that the *FLT3* mutation would more prevalent in the unfavorable prognostic group than in the favorable prognostic group. This might be due to the proliferative nature of this mutation, which contributes to cellular proliferation, at least in the short term.

The prognostic scoring system for D30 survival after GT in this study revealed that impaired organ function, including hematopoiesis and liver and renal function, was related to D30 survival. Eight factors were associated with D30 survival including secondary AML and the LDH level. The platelet count, PT, and mean AUC-WBC could represent hematopoiesis; the bilirubin, ALT, and PT values could reflect liver function; and phosphorus and BUN levels might reflect renal function.

The clinical outcomes of patients with AML vary widely and are affected by several factors. Important known factors include cytogenetics, molecular abnormalities, and age at diagnosis [31]. In addition, a short bone marrow recovery time, estimated by the platelet count and neutrophil recovery time after induction chemotherapy, is associated with better clinical outcomes [32]. In our study, the D30 survivor and nonsurvivor groups did not show significant differences in the number of RBCs or platelet transfusions. However, it would have been better if we adjusted for these confounders, which may have statistically affected our results.

The international collection of patient data in a registry is ongoing [33]. We expect that this international registry of GT could accumulate patient data for analysis and elucidate the effect of GT not only in AML but also in other hematologic diseases [34].

This study has some limitations, including the lack of a control group. Additionally, propensity score matching could have been an alternative method; however, establishing a control group that matched patients undergoing GT was not feasible. Moreover, antimicrobial susceptibility tests were not conducted and the pathogenicity of microbes was not fully evaluated. Lastly, arterial pressure and blood gas analysis data, which may have affected D30 prognosis, were not collected.

In conclusion, this study identified significant prognostic parameters for GT efficacy and developed a scoring system based on these parameters. Our study findings may contribute to the estimation of patient prognosis after GT and future randomized control trials.

## Supporting information

S1 File (PDF)

## References

[1] Drewniak A, Kuijpers TW. Granulocyte transfusion therapy: randomization after all? Haematologica. 2009;94(12):1644–8. doi: 10.3324/haematol.2009.013680 ; PubMed Central PMCID: PMC2791939. 19996116 PMC2791939

[2] Bishton M, Chopra R. The role of granulocyte transfusions in neutropenic patients. Br J Haematol. 2004;127(5):501–8. doi: 10.1111/j.1365-2141.2004.05221.x . 15566353

[3] West KA, Gea-Banacloche J, Stroncek D, Kadri SS. Granulocyte transfusions in the management of invasive fungal infections. Br J Haematol. 2017;177(3):357–74. Epub 20170314. doi: 10.1111/bjh.14597 ; PubMed Central PMCID: PMC5403628. 28295178 PMC5403628

[4] Valentini CG, Farina F, Pagano L, Teofili L. Granulocyte Transfusions: A Critical Reappraisal. Biol Blood Marrow Transplant. 2017;23(12):2034–41. Epub 20170807. doi: 10.1016/j.bbmt.2017.07.029 . 28797785

[5] Price TH, Boeckh M, Harrison RW, McCullough J, Ness PM, Strauss RG, et al. Efficacy of transfusion with granulocytes from G-CSF/dexamethasone-treated donors in neutropenic patients with infection. Blood. 2015;126(18):2153–61. Epub 20150902. doi: 10.1182/blood-2015-05-645986 ; PubMed Central PMCID: PMC4626256. 26333778 PMC4626256

[6] Cugno C, Deola S, Filippini P, Stroncek DF, Rutella S. Granulocyte transfusions in children and adults with hematological malignancies: benefits and controversies. J Transl Med. 2015;13:362. Epub 20151116. doi: 10.1186/s12967-015-0724-5 ; PubMed Central PMCID: PMC4647505. 26572736 PMC4647505

[7] Estcourt LJ, Stanworth S, Doree C, Blanco P, Hopewell S, Trivella M, et al. Granulocyte transfusions for preventing infections in people with neutropenia or neutrophil dysfunction. Cochrane Database Syst Rev. 2015;2015(6):Cd005341. Epub 20150629. doi: 10.1002/14651858.CD005341.pub3 ; PubMed Central PMCID: PMC4538863. 26118415 PMC4538863

[8] Netelenbos T, Massey E, de Wreede LC, Harding K, Hamblin A, Sekhar M, et al. The burden of invasive infections in neutropenic patients: incidence, outcomes, and use of granulocyte transfusions. Transfusion. 2019;59(1):160–8. Epub 20181101. doi: 10.1111/trf.14994 ; PubMed Central PMCID: PMC7379528. 30383912 PMC7379528

[9] Marfin AA, Price TH. Granulocyte transfusion therapy. J Intensive Care Med. 2015;30(2):79–88. Epub 20130805. doi: 10.1177/0885066613498045 . 23920161

[10] Németh T, Sperandio M, Mócsai A. Neutrophils as emerging therapeutic targets. Nat Rev Drug Discov. 2020;19(4):253–75. Epub 20200122. doi: 10.1038/s41573-019-0054-z . 31969717

[11] Kolaczkowska E, Kubes P. Neutrophil recruitment and function in health and inflammation. Nat Rev Immunol. 2013;13(3):159–75. doi: 10.1038/nri3399 . 23435331

[12] Tecchio C, Cassatella MA. Neutrophil-derived chemokines on the road to immunity. Semin Immunol. 2016;28(2):119–28. Epub 20160414. doi: 10.1016/j.smim.2016.04.003 ; PubMed Central PMCID: PMC7129466. 27151246 PMC7129466

[13] Garg A, Gupta A, Mishra A, Singh M, Yadav S, Nityanand S. Role of granulocyte transfusions in combating life-threatening infections in patients with severe neutropenia: Experience from a tertiary care centre in North India. PLoS One. 2018;13(12):e0209832. Epub 20181227. doi: 10.1371/journal.pone.0209832 ; PubMed Central PMCID: PMC6307785. 30589898 PMC6307785

[14] Scheff JD, Almon RR, Dubois DC, Jusko WJ, Androulakis IP. Assessment of pharmacologic area under the curve when baselines are variable. Pharm Res. 2011;28(5):1081–9. Epub 20110114. doi: 10.1007/s11095-010-0363-8 ; PubMed Central PMCID: PMC3152796. 21234658 PMC3152796

[15] Urso R, Blardi P, Giorgi G. A short introduction to pharmacokinetics. Eur Rev Med Pharmacol Sci. 2002;6(2–3):33–44. . 12708608

[16] Quillen K, Wong E, Scheinberg P, Young NS, Walsh TJ, Wu CO, et al. Granulocyte transfusions in severe aplastic anemia: an eleven-year experience. Haematologica. 2009;94(12):1661–8. doi: 10.3324/haematol.2009.010231 ; PubMed Central PMCID: PMC2791947. 19996117 PMC2791947

[17] Jung J, Cho BS, Kim HJ, Han E, Jang W, Han K, et al. Reclassification of Acute Myeloid Leukemia According to the 2016 WHO Classification. Ann Lab Med. 2019;39(3):311–6. doi: 10.3343/alm.2019.39.3.311 ; PubMed Central PMCID: PMC6340847. 30623623 PMC6340847

[18] Yoon JH, Kim HJ, Park SS, Jeon YW, Lee SE, Cho BS, et al. Clinical Outcome of Autologous Hematopoietic Cell Transplantation in Adult Patients with Acute Myeloid Leukemia: Who May Benefit from Autologous Hematopoietic Cell Transplantation? Biol Blood Marrow Transplant. 2017;23(4):588–97. Epub 20170112. doi: 10.1016/j.bbmt.2017.01.070 . 28089879

[19] Lee DG, Kim SH, Kim SY, Kim CJ, Park WB, Song YG, et al. Evidence-based guidelines for empirical therapy of neutropenic fever in Korea. Korean J Intern Med. 2011;26(2):220–52. Epub 20110601. doi: 10.3904/kjim.2011.26.2.220 ; PubMed Central PMCID: PMC3110859. 21716917 PMC3110859

[20] Cho SY, Lee DG, Choi SM, Choi JK, Lee HJ, Kim SH, et al. Posaconazole for primary antifungal prophylaxis in patients with acute myeloid leukaemia or myelodysplastic syndrome during remission induction chemotherapy: a single-centre retrospective study in Korea and clinical considerations. Mycoses. 2015;58(9):565–71. Epub 20150727. doi: 10.1111/myc.12357 . 26214656

[21] Lee H, Park S, Yoon JH, Cho BS, Kim HJ, Lee S, et al. The factors influencing clinical outcomes after leukapheresis in acute leukaemia. Sci Rep. 2021;11(1):6426. Epub 20210319. doi: 10.1038/s41598-021-85918-8 ; PubMed Central PMCID: PMC7979875. 33742034 PMC7979875

[22] Robin X, Turck N, Hainard A, Tiberti N, Lisacek F, Sanchez JC, et al. pROC: an open-source package for R and S+ to analyze and compare ROC curves. BMC Bioinformatics. 2011;12:77. Epub 20110317. doi: 10.1186/1471-2105-12-77 ; PubMed Central PMCID: PMC3068975. 21414208 PMC3068975

[23] Therneau T. A package for survival analysis in R. (R package version 3.1–12.)^(eds) Book A Package for Survival Analysis in R R package version. 2020:3.1–12.

[24] Kassambara A, Kosinski M, Biecek P, Fabian S. survminer: Drawing Survival Curves using ‘ggplot2’. R package version 03. 2017;1.

[25] Wickham H, Grolemund G. R for data science: Import. Tidy, transform, visualize, and model data. 2017;1.

[26] Poubelle PE, Pagé N, Longchamps MP, Sampaio Moura N, Beck DB, Aksentijevich I, et al. The use of leukocytes’ secretome to individually target biological therapy in autoimmune arthritis: a case report. Clin Transl Med. 2019;8(1):19. Epub 20190605. doi: 10.1186/s40169-019-0236-7 ; PubMed Central PMCID: PMC6548783. 31165299 PMC6548783

[27] Blanch-Ruiz MA, Ortega-Luna R, Martínez-Cuesta M, Álvarez Á. The Neutrophil Secretome as a Crucial Link between Inflammation and Thrombosis. Int J Mol Sci. 2021;22(8). Epub 20210417. doi: 10.3390/ijms22084170 ; PubMed Central PMCID: PMC8073391. 33920656 PMC8073391

[28] Kadri SS, Remy KE, Strich JR, Gea-Banacloche J, Leitman SF. Role of granulocyte transfusions in invasive fusariosis: systematic review and single-center experience. Transfusion. 2015;55(9):2076–85. Epub 20150409. doi: 10.1111/trf.13099 ; PubMed Central PMCID: PMC4573241. 25857209 PMC4573241

[29] Busca A, Cesaro S, Teofili L, Delia M, Cattaneo C, Criscuolo M, et al. SEIFEM 2017: from real life to an agreement on the use of granulocyte transfusions and colony-stimulating factors for prophylaxis and treatment of infectious complications in patients with hematologic malignant disorders. Expert Rev Hematol. 2018;11(2):155–68. Epub 20180103. doi: 10.1080/17474086.2018.1420472 . 29285951

[30] Rosales C. Neutrophil: A Cell with Many Roles in Inflammation or Several Cell Types? Front Physiol. 2018;9:113. Epub 20180220. doi: 10.3389/fphys.2018.00113 ; PubMed Central PMCID: PMC5826082. 29515456 PMC5826082

[31] Döhner H, Estey E, Grimwade D, Amadori S, Appelbaum FR, Büchner T, et al. Diagnosis and management of AML in adults: 2017 ELN recommendations from an international expert panel. Blood. 2017;129(4):424–47. Epub 20161128. doi: 10.1182/blood-2016-08-733196 ; PubMed Central PMCID: PMC5291965. 27895058 PMC5291965

[32] Çiftçiler R, Haznedaroğlu İ C, Sayınalp N, Özcebe O, Aksu S, Demiroğlu H, et al. The Impact of Early Versus Late Platelet and Neutrophil Recovery after Induction Chemotherapy on Survival Outcomes of Patients with Acute Myeloid Leukemia. Turk J Haematol. 2020;37(2):116–20. Epub 20190902. doi: 10.4274/tjh.galenos.2019.2019.0154 ; PubMed Central PMCID: PMC7236414. 31475513 PMC7236414

[33] Pagano MB, Morton S, Cohn CS, Gross S, Kutner J, Lewin A, et al. An International Registry of Granulocyte Transfusions. Transfus Med Hemother. 2018;45(5):318–22. Epub 20180824. doi: 10.1159/000492629 ; PubMed Central PMCID: PMC6257207. 30498409 PMC6257207

[34] Gale RP, Schiffer CA, Lazarus HM. Granulocyte transfusions in haematopoietic cell transplants and leukaemia: the phoenix or beating a dead horse? Bone Marrow Transplant. 2021;56(9):2046–9. Epub 20210703. doi: 10.1038/s41409-021-01399-3 . 34218266

