# Supplementary material for: Risk stratification by 30-day prognostic factors of clinical outcomes after granulocyte transfusion in acute myeloid leukemia: A single-center retrospective study
Source: PLoS One. 2022 Aug 30;17(8):e0273827. doi: 10.1371/journal.pone.0273827 (PMC9426873; doi:10.1371/journal.pone.0273827)

SUPPLEMENTARY MATERIALS

Risk stratification by 30 day prognostic factors after granulocyte transfusion in acute myeloid leukemia: a single center retrospective study

Jaeeun Yoo[1], Hyung Suk Cho[2,3], Jae-Ho Yoon[4], Byung Sik Cho[4], Hee-Je Kim[4], Dong-Gun Lee[5], Dong Wook Jekarl[2,3,6*], Myungshin Kim[2], Eun-Jee Oh[2,6], Yeon-Joon Park[2], Yonggoo Kim[2]

[1]Department of Laboratory Medicine, Incheon St. Mary's Hospital, College of Medicine, The Catholic University of Korea, Seoul, Korea

[2]Department of Laboratory Medicine, Seoul St. Mary's Hospital, College of Medicine, The Catholic University of Korea, Seoul, Korea

[3]Department of Laboratory Medicine, Aphresis Unit, Seoul St. Mary's Hospital, College of Medicine, The Catholic University of Korea, Seoul, Korea

[4]Department of Internal Medicine, Catholic Hematology Hospital, Seoul St. Mary's Hospital, College of Medicine, The Catholic University of Korea, Seoul, Korea

[5]Division of Infectious Diseases, Department of Internal Medicine, Seoul St. Mary's Hospital, College of Medicine, The Catholic University of Korea, Seoul, Korea

[6]Research and Development Institute for In Vitro Diagnostic Medical Devices, College of Medicine, The Catholic University of Korea, Seoul, Korea

SUPPLEMENTARY TABLES

Supplementary Table S1. Threshold of laboratory that showed largest area under the receiver operating characteristic curve for D30 survival

	Threshold	Specificity	Sensitivity
WBC (10^9/L)	1.14	0.68	0.47
WBC-AUC	10	0.72	0.46
Neutrophil (%)	32.7	0.79	0.40
Lymphocyte (%)	18.8	0.82	0.43
Hg (g/L)	84.5	0.90	0.27
PLT (10^9/L)	24.5	0.90	0.28
RDW (%)	13.8	0.46	0.81
PT (INR)	1.35	0.82	0.60
aPTT (sec)	40.2	0.75	0.45
Creatinine (umol/L)	75.2	0.78	0.59
Protein (g/L)	52.5	0.74	0.47
Albumin (g/L)	25.5	0.90	0.28
Bilirubin (umol/L)	50.5	0.76	0.61
Direct bilirubin (umol/L)	28.2	0.71	0.64
AST (ukat/L)	0.41	0.68	0.63
ALT (ukat/L)	0.78	0.93	0.25
L DH (ukat/L)	9.93	0.85	0.59
Phosphorus (mmol/L)	1.21	0.87	0.35
Chloride (mmol/L)	107.6	0.90	0.33
Magnesium (mmol/L)	0.84	0.57	0.61
ESR (mm/hr)	4.5	0.99	0.17
CRP (mg/L)	216	0.84	0.51

Abbreviation: WBC, white blood cell; AUC, area under the curve; Hg, hemoglobin; PLT, platelet; RDW, red blood cell distribution width; PT INR, prothrombin time international normalized ratio; aPTT, activated thromboplastin time; AST, aspartate aminotransferase; ALT, alanine aminotransferase; LDH, lactate dehydrogenase, ESR, erythrocyte sedimentation rate; CRP, C reactive protein

Supplementary Table S2. Kaplan Meier survival analysis

		records	max	start	event	rmean	se rmean	median	95% CI, low	95% CI, high	P value
Secondary	Low	178	178	178	63	22.1	0.9	NA	NA	NA	0.02
AML	High	22	22	22	13	16.3	2.6	12.5	7	NA	
Microbe	0	55	55	55	15	23.3	1.5	NA	NA	NA	0.06
Growth	1 time	67	67	67	22	22.5	1.4	NA	NA	NA	
frequency	2 time	97	97	97	45	19.8	1.2	NA	19	NA	
WBC	Low	79	79	79	35	19.4	1.4	NA	15	NA	0.02
	High	132	132	132	4	23.6	0.9	NA	NA	NA	
AUC	Low	76	76	76	37	18.0	1.5	NA	10	NA	0.002
	High	142	142	142	44	23.5	0.9	NA	NA	NA	
Neutr	Low	59	59	59	30	17.7	1.7	26	10	NA	8.00E-04
	High	152	152	152	45	23.7	0.8	NA	NA	NA	
Lymph	Low	57	57	57	32	16.9	1.7	18	6	NA	2.00E-05
	High	154	154	154	43	23.9	0.8	NA	NA	NA	
Hg	Low	34	34	34	20	15.1	2.3	11	2	NA	7.00E-05
	High	177	177	177	55	23.4	0.8	NA	NA	NA	
PLT	Low	35	35	35	21	14.8	2.2	10	3	NA	4.00E-05
	High	176	176	176	54	23.5	0.8	NA	NA	NA	
RDW	Low	55	55	55	10	26.3	1.1	NA	NA	NA	0.001
	High	95	95	95	42	20.0	1.2	NA	17	NA	
PT INR	Low	141	141	141	30	25.9	0.7	NA	NA	NA	1.00E-12
	High	70	70	70	45	14.3	1.5	7.5	5	26	
aPTT	Low	143	143	143	41	24.2	0.8	NA	NA	NA	4.00E-04
	High	68	68	68	34	17.5	1.6	17	7	NA	
Creatinine	Low	137	137	137	31	25.2	0.8	NA	NA	NA	7.00E-09
	High	74	74	74	44	16.1	1.5	14	7	NA	
Protein	Low	70	70	70	35	18.0	1.5	26	11	NA	5.00E-04
	High	141	141	141	40	24.1	0.9	NA	NA	NA	
Albumin	Low	35	35	35	21	15.7	2.2	15	4	NA	1.00E-04
	High	176	176	176	54	23.3	0.8	NA	NA	NA	
Bilirubin	Low	133	133	133	29	25.3	0.8	NA	NA	NA	6.00E-09
	High	78	78	78	46	16.5	1.4	14.5	10	NA	
D.bilirubin	Low	124	124	124	27	25.3	0.8	NA	NA	NA	2.00E-07
	High	87	87	87	48	17.3	1.3	17	11	NA	
AST	Low	120	120	120	28	24.8	0.9	NA	NA	NA	1.00E-05
	High	91	91	91	47	18.4	1.3	25	12	NA	
ALT	Low	183	183	183	56	23.4	0.8	NA	NA	NA	3.00E-06
	High	28	28	28	19	13.4	2.3	7	2	NA	
LDH	Low	146	146	146	31	25.3	0.8	NA	NA	NA	2.00E-12
	High	65	65	65	44	14.6	1.5	11	7	21	
Phos	Low	167	167	167	49	23.6	0.8	NA	NA	NA	5.00E-05
	High	44	44	44	26	16.2	1.9	13	6	NA	
Cl	Low	131	131	131	41	22.9	1.0	NA	NA	NA	1.00E-04
	High	30	30	30	20	15.1	2.2	11.5	6	NA	
Mg	Low	106	106	106	29	23.8	1.0	NA	NA	NA	0.01
	High	105	105	105	46	20.2	1.2	NA	21	NA	
ESR	Low	13	13	13	12	6.5	2.6	3	1	NA	5.00E-12
	High	189	189	189	59	23.2	0.8	NA	NA	NA	
CRP	Low	151	151	151	37	24.6	0.8	NA	NA	NA	8.00E-09

High	60	60	60	38	15.6	1.6	11.5	7	NA

Abbreviation: WBC, white blood cell; AUC, area under the curve; Neutr, neutrophil, Lymph, lymphocyte; Hg, hemoglobin; PLT, platelet; RDW, red blood cell distribution width; PT INR, prothrombin time international normalized ratio; aPTT, activated thromboplastin time; AST, aspartate aminotransferase; ALT, alanine aminotransferase; LDH, lactate dehydrogenase, Phos, phosphorus; Cl, chloride; Mg, magnesium; ESR, erythrocyte sedimentation rate; CRP, C reactive protein

Supplementary Table S3. Univariate analysis for 30 day mortality prediction

	β coefficient	*P*	HR (95% CI)
Age			
age < 60	0 (reference)	0.184	1 (reference)
age ≥ 60	0.331		1.392 (0.854-2.270)
Sex			
female	0 (reference)	0.482	1 (reference)
male	0.159		1.173 (0.752-1.828)
Secondary AML			
NO	0 (reference)	0.023	1 (reference)
YES	0.696		2.005 (1.103-3.646)
AML, MRC			
NO	0 (reference)	0.047	1 (reference)
YES	0.541		1.718 (1.007-2.931)
Relapse			
NO	0 (reference)	0.663	1 (reference)
YES	0.113		1.120 (0.672-1.867)
Reinduction CTx			
NO	0 (reference)	0.328	1 (reference)
YES	0.220		1.247 (0.802-1.939)
FLT3 mutation			
NO	0 (reference)	0.328	1 (reference)
YES	-0.368		0.692 (0.343-1.396)
Baalc gene expression			
< 7.04	0 (reference)	0.568	1 (reference)
≥ 7.04	0.159		1.172 (0.680-2.022)
WT1 gene expression			
< 0.17	0 (reference)	0.568	1 (reference)
≥ 0.17	0.227		1.338 (0.835-2.145)
GT (unit)			
< 6	0 (reference)	0.944	1 (reference)
≥ 6	-0.058		0.944 (0.605-1.472)
Red blood cell transfusion (unit)			
< 5	0 (reference)	0.335	1 (reference)
≥ 5	0.341		1.406 (0.703-2.810)
Platelet transfusion (unit)			
< 13	0 (reference)	0.101	1 (reference)
≥ 13	0.372		1.450 (0.930-2.261)
White blood cell (10^9/L)			
< 1.13	0 (reference)	0.031	1 (reference)
≥ 1.13	-0.501		0.607 (0.385

Hemoglobin (g/L)			
< 84	0 (reference)	0.031	1 (reference)
≥ 84	-0.986		0.373 (0.221-0.629)
Platelet (10^9/L)			
< 30	0 (reference)	0.001	1 (reference)
≥ 30	-0.788		0.455 (0.280-0.739)
mean AUC-WBC (area/GT)			
< 3.03	0 (reference)	0.004	1 (reference)
≥ 3.03	-1.236		0.291 (0.126-0.668)
PT (INR)			
< 1.3	0 (reference)	<0.001	1 (reference)
≥ 1.3	1.435		4.201 (2.292-6.808
aPTT (sec)			
< 40	0 (reference)	0.001	1 (reference)
≥ 40	0.764		2.146 (1.361-3.386)
Blood urea nitrogen (mmol/L)			
< 12	0 (reference)	<0.001	1 (reference)
≥ 12	1.623		5.068 (3.110-8.259)
Creatinine (umol/L)			
< 53.4	0 (reference)	<0.001	1 (reference)
≥ 53.4	1.264		3.541 (2.102-5.965)
Protein (g/L)			
< 53	0 (reference)	0.006	1 (reference)
≥ 55	-0.687		0.503 (0.308-0.822)
Albumin (g/L)			
< 25.3	0 (reference)	0.001	1 (reference)
≥ 25.3	-0.890		0.411 (0.239-0.706)
Bilirubin (mmol/L)			
< 51.3	0 (reference)	<0.001	1 (reference)
≥ 51.3	1.276		3.581 (2.246-5.710)
AST (ukat/L)			
< 0.42	0 (reference)	<0.001	1 (reference)
≥ 0.42	1.038		2.824 (1.767-4.513)
ALT (ukat/L)			
< 0.76	0 (reference)	<0.001	1 (reference)
≥ 0.76	1.159		3.186 (1.888-5.376)
Sodium (mmol/L)			
< 141.4	0 (reference)	<0.001	1 (reference)
≥ 141.4	0.577		1.781 (1.111-2.854)
Potassium (mmol/L)			
< 3.5	0 (reference)	0.258	1 (reference)
≥ 3.5	0.262		1.299 (0.826-2.043)

Phosphorus (mmol/L)			
< 1.22	0 (reference)	<0.001	1 (reference)
≥ 1.22	0.986		2.681 (1.590-4.519)
Calcium (mmol/L)			
< 1.82	0 (reference)	0.001	1 (reference)
≥ 1.82	-1.036		0.355 (0.195-0.646)
Uric acid (mol/L)			
< 0.16	0 (reference)	<0.001	1 (reference)
≥ 0.16	1.017		2.764 (1.747-4.373)
LDH (ukat/L)			
< 9.9	0 (reference)	<0.001	1 (reference)
≥ 9.9	1.463		4.320 (2.724-6.851)
CRP (mg/L)			
< 217	0 (reference)	<0.001	1 (reference)
≥ 217	0.986		2.681 (1.703-4.223)
ESR (mm/hr)			
< 19	0 (reference)	0.010	1 (reference)
≥ 19	-0.635		0.530 (0.328-0.853)

AML, acute myeloid leukemia; MRC, myelodysplasia relatd changes; CTx, chemotherapy; AUC, area under the curve; WBC, white blood cell; GT, granulocyte transfusion; RBC, red blood cell; RDW, red blood cell distriubion width; PT, prothrombin time; aPTT, activated partial thromboplastin time; AST, aspartate aminotransferate; ALT, alanine aminotransferate; ; LDH, lactate dehydrogenase; CRP, c-reactive protein; ESR, erythrocyte sedimentation rate

Supplementary Table S4. Baseline data of Secondary AML patients

Age	Underlying Disease	Diagnosis	D30 survival
49	AA	AML, NOS	Nonsurvivor
53	AA	AML, NOS	Survivor
22	AA	AML, NOS	Survivor
46	atypical CML	AML with MRC	Survivor
53	MPNU	AML with KMT2A	Nonsurvivor
51	CMMoL	AML with NPM1 gene mutation	Nonsurvivor
64	CNL	AML, NOS	Nonsurvivor
58	ET	AML, NOS	Nonsurvivor
35	MDS	AML with MRC	Survivor
64	MDS	AML, NOS	Nonsurvivor
40	MDS	AML, NOS	Survivor
69	MDS	AML with MRC	Nonsurvivor
57	MDS	AML with MRC	Survivor
51	MDS	AML with MRC	Survivor
36	MDS	AML with MRC	Survivor
43	MDS	AML with KMT2A abnormalities	Survivor
63	MDS	AML with MRC	Nonsurvivor
30	MDS	AML with MRC	Survivor
47	MDS	AML with MRC	Nonsurvivor
65	MDS	AML with MRC	Survivor
69	MDS/MPN	AML with MRC	Nonsurvivor
58	MPNU	AML, NOS	Survivor
67	MPNU	AML with MRC	Nonsurvivor
24	MPNU	AML, NOS	Nonsurvivor
59	PMF	AML, NOS	Nonsurvivor
65	MPNU	AML with MRC	Survivor

AA, aplastic aneia; AML, acute myeloid leukemia; NOS, not otherwise specific; MRC, myelodysplastic related changes; CML, chronic myeloid leukemia; MPNU, myeloproliferative neoplasm, unclassifiable; CMMoL, chronic myelomonocytic leukemia; CNL, chronic neutrophilic leukemia; ET, essential thrombocythemia; MDS, myelodysplastic syndrome; PMF, primary myelofibrosis

Supplementary Table S5. Recovered microbes from cultured specimen

	Blood	Catheter	Sputum	Urine	Stool	Others
Gram positive						
Bacillus spp.	1	0	0	0	0	1
Cutibacterium acnes	1	0	0	0	0	0
Coagulase negative staphylococcus	1	0	0	3	0	0
Clostridium difficle	0	0	0	0	24	0
Clostridium perfringens	0	1	0	0	0	0
Clostridium tertium	0	1	0	0	0	0
Enterococcus avium	0	0	0	0	0	1
Enterococcus faecalis	5	0	0	0	0	1
Enterococcus faecium	45	9	0	9	0	3
Enterococcus gallinarum	1	0	0	0	0	0
Enterococcus spp.	0	0	0	2	0	0
Staphylococcus aureus	8	5	4	0	0	3
Staphylococcus epidermidis	8	5	1	0	0	1
Staphylococcus hominis	4	2	0	0	0	0
Staphylococcus haemolyticus	1	0	0	0	0	0
Streptococcus agalactiae	0	1	0	0	0	0
Streptococcus miits, oralis	4	0	0	0	0	0
Streptococcus pneumoniae	0	0	1	0	0	0
Streptococcus viridans	14	8	0	0	0	0
Subtotal	93	32	6	14	24	10
Gram negative						
Acinetobacter baumannii	0	0	4	0	0	2
Acinetobacter lwoffii	0	0	1	0	0	0
Burkholderia cepacia	1	1	0	0	0	0
Chryseobacterium indoogenes	0	0	1	0	0	0
E. coli	23	17	2	4	0	1
Enterobacter aerogenase	0	1	0	0	0	0
Enterobacter cloacae	1	1	0	0	0	0
Eikenella corrodens	0	1	0	0	0	0
Fusobacterium nucleatum	1	0	0	0	0	0
Morganella morganii	0	1	0	0	0	0
Klebsiella pneumoniae	11	7	0	1	0	0
Klebsiella oxytoca	0	0	0	1	0	0
Pseudomonas aeruginosa	5	1	2	0	1	1
Stenotrophomonas maltophilia	1	2	4	0	0	2
Subtotal	43	32	14	6	1	6
Fungus, yeast						
Aspergillus fumigatus	0	0	2	0	0	0

Aspergillus terrueus	0	0	1	0	0	0
Candida crusei	1	0	0	0	0	0
Candida glabrata	1	0	0	0	0	0
Candida parasilosis	1	0	0	0	0	1
Rodotorula mucilaginosa	2	0	0	0	0	0
Yeast spp.	1	0	0	0	0	0
Subtotal	6	0	3	0	0	1
Total	142	64	23	20	25	17

Supplementary Table S6. Baseline characteristics of laboratory data

	N	Survivor (n=137)	Nonsurvivor (n=82)	P value
WBC (10^9/L)	211	1.86± 1.37	1.73± 1.69	NS
Neutrophil (%)	211	46.9± 20.2	38.1± 25.8	0.015
Lymphocyte (%)	211	37.9± 22.2	26.6± 22.4	<0.001
AUC-WBC	204	14.4±20.8	8.5±11.7	0.006
RBC (10^{12}/L)	211	3.38± 0.52	3.28± 0.64	NS
Hemoglobin (g/L)	211	102± 16.4	98.7± 19.7	NS
Hematocrit (%)	211	29.6± 4.81	28.7± 5.66	NS
RBC distribution Width (%)	150	14.0± 1.18	14.6± 1.15	NS
Platelet (10^9/L)	211	78.7± 49.2	64.9± 53.2	NS
Prothrombin time (sec)	211	13.6± 1.78	16.5±4.01	<0.001
Partial prothrombin time (sec)	211	37.2±8.75	41.1±11.5	0.008
Glucose (mmol/L)	195	8.77±2.89	9.16±3.05	NS
Creatinine (umol/L)	211	59.5±38.9	89.9±60.2	<0.001
Protein (g/L)	211	56.8±6.9	54.0±8.4	0.026
Albumin (g/L)	211	29.1±3.1	27.9±4.0	0.029
Total bilirubin (umol/L)	211	50.55±69.4	96.9±83.9	<0.001
Direct bilibrubin (umol/L)	211	36.6±57.3	69.7±66.2	<0.001
Asparate transaminase (ukat/L)	211	0.53±1.3	2.82±5.95	0.021
Alanine transaminase (ukat/L)	211	0.43±0.43	1.14±3.14	0.034
Calcium (mmol/L)	211	1.98±0.11	1.96±0.16	NS
Potassium (mmol/L)	211	3.43±0.45	3.48±0.56	NS
Phosphorus (mmol/L)	211	0.94±0.25	1.10±0.38	0.003
Sodium (mmol/L)	211	139±4.68	143±7.58	<0.001
Chloride (mmol/L)	161	102±4.53	104±6.61	NS
Magnesium (mmol/L)	211	0.83±0.1	0.87±0.15	NS
Lactate dehydrogenase (ukat/L)	211	8.12±8.20	17.89±22.71	<0.001
Amylase (ukat/L)	211	1.15±1.19	1.31±1.36	NS
ESR (mm/hr)	201	38±22	32±24	NS
C-reactive protein (mg/L)[a]	211	157±71.6	219±79.6	<0.001

[a]All the analysis was perform by Mann-Whitey U test except for C-reactive protein

WBC, white blood cell; AUC, area under the curve; ESR, erythrocyte sedimentation rate

SUPPLEMENTARY FIGURES

Supplementary Figure S1. Flow chart of patient enrollment

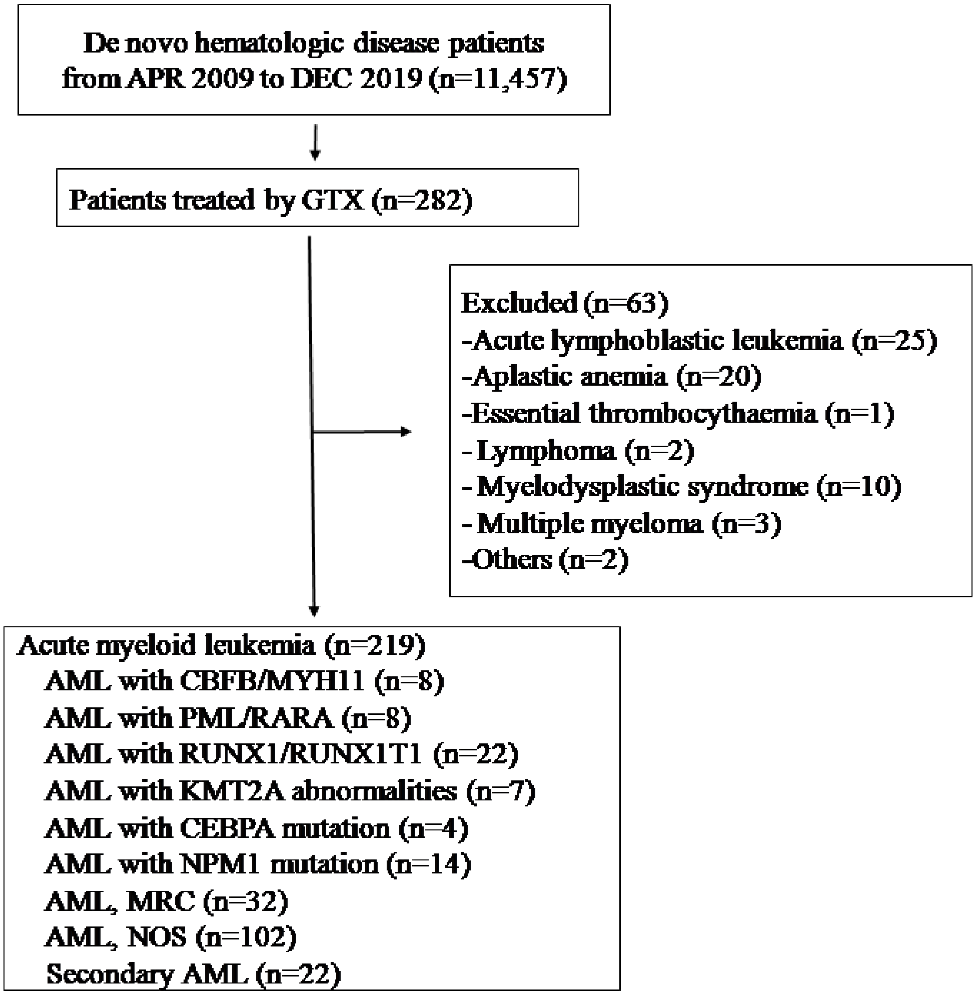

GTX, granulocyte transfusion; AML, acute myeloid leukemia

Supplementary Figure S2. Area under the curve (AUC) of white blood cell (WBC) before granulocyte transfusion among day 30 survivors. Area of light blue color under the WBC count was calculated using trapezoidal method[a] .

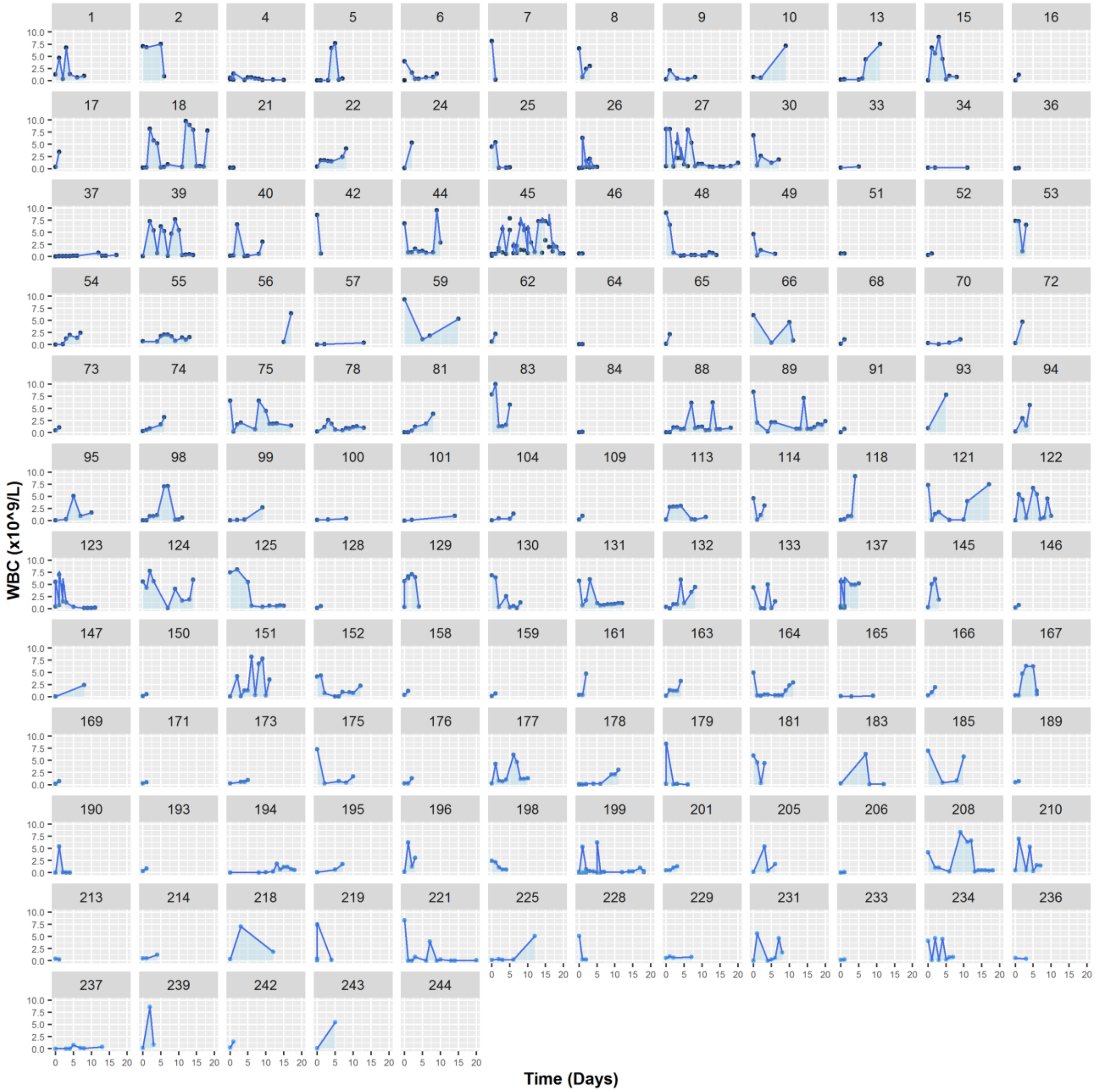

Supplementary Figure S3. Area under the curve (AUC) of white blood cell (WBC) before granulocyte transfusion among day 30 nonsurvivors. Area of light blue color under the WBC count was calculated using trapezoidal method.

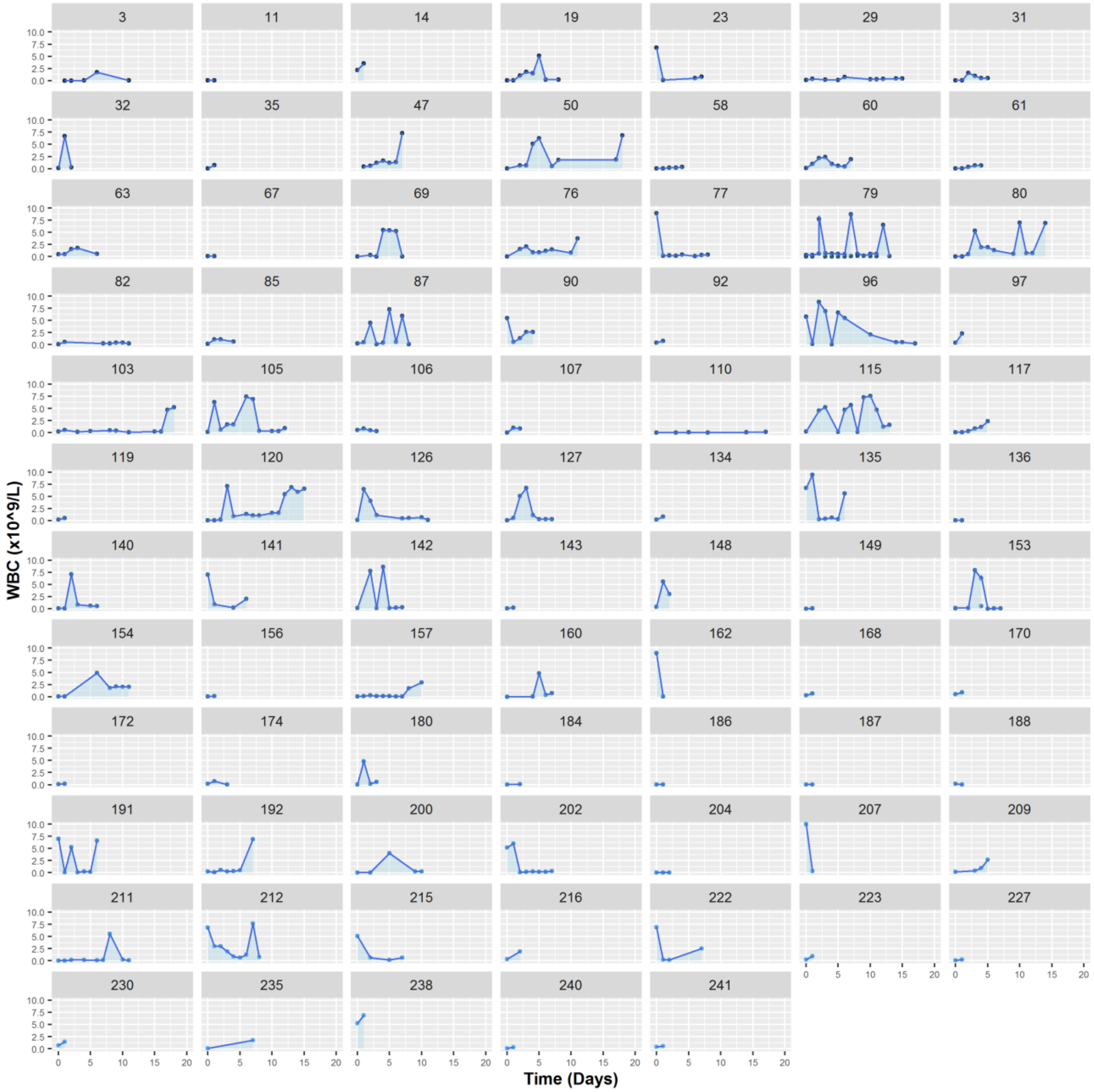

Supplementary Figure S4. Kaplan Meier survival analysis of clinical and laboratory parameters. All of the comparison of survival curve curved showed P<0.05 except for microbial growth frequency (P=0.06). Shade around line are 95% confidence interval of survival curve. 2ndAML, Secondary AML; M.growth, microbial growth frequency

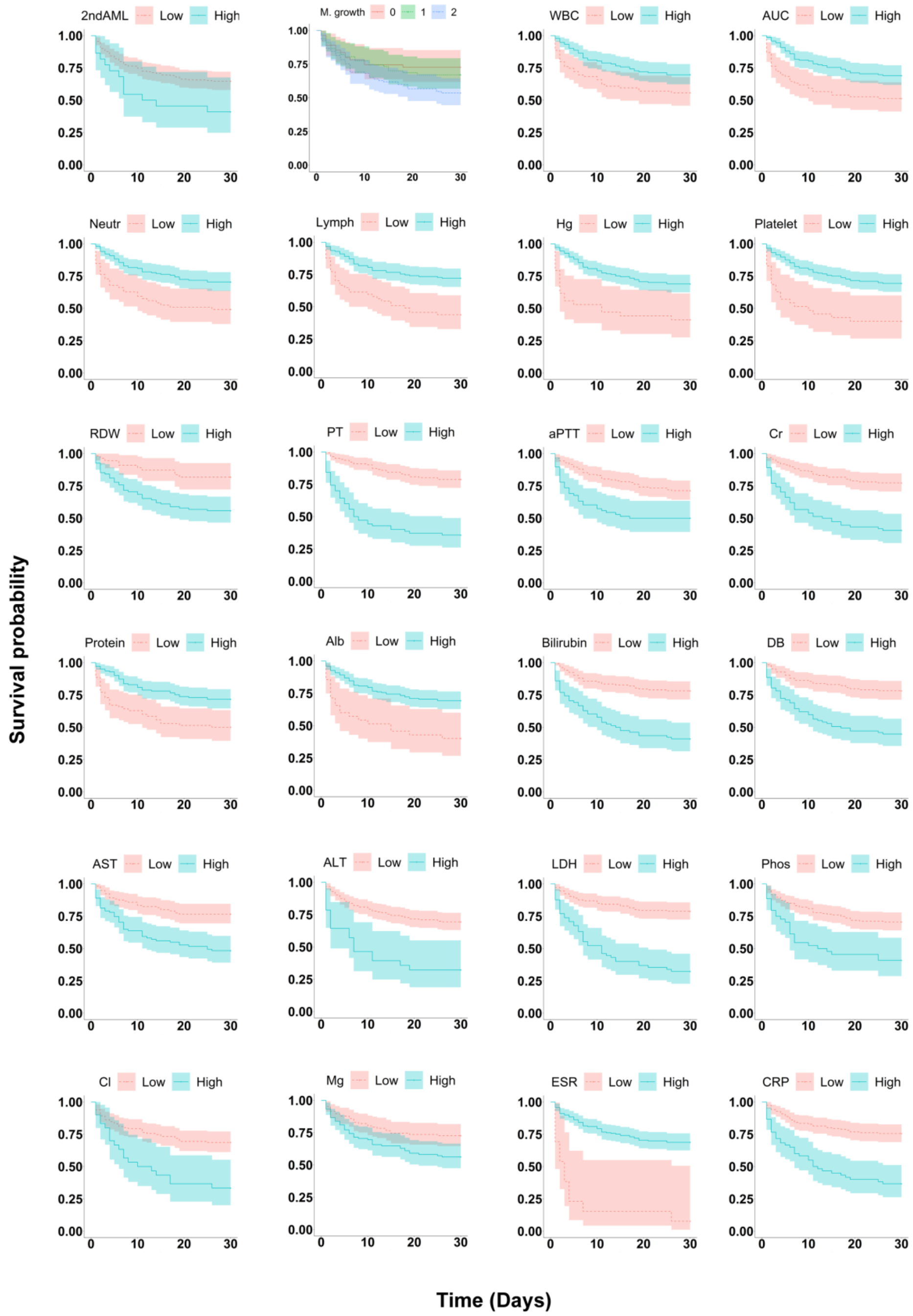

Supplementary Figure S5. Laboratory parameters before and after GT for survivor (group=0) and nonsurivor (group=1) at day 30.

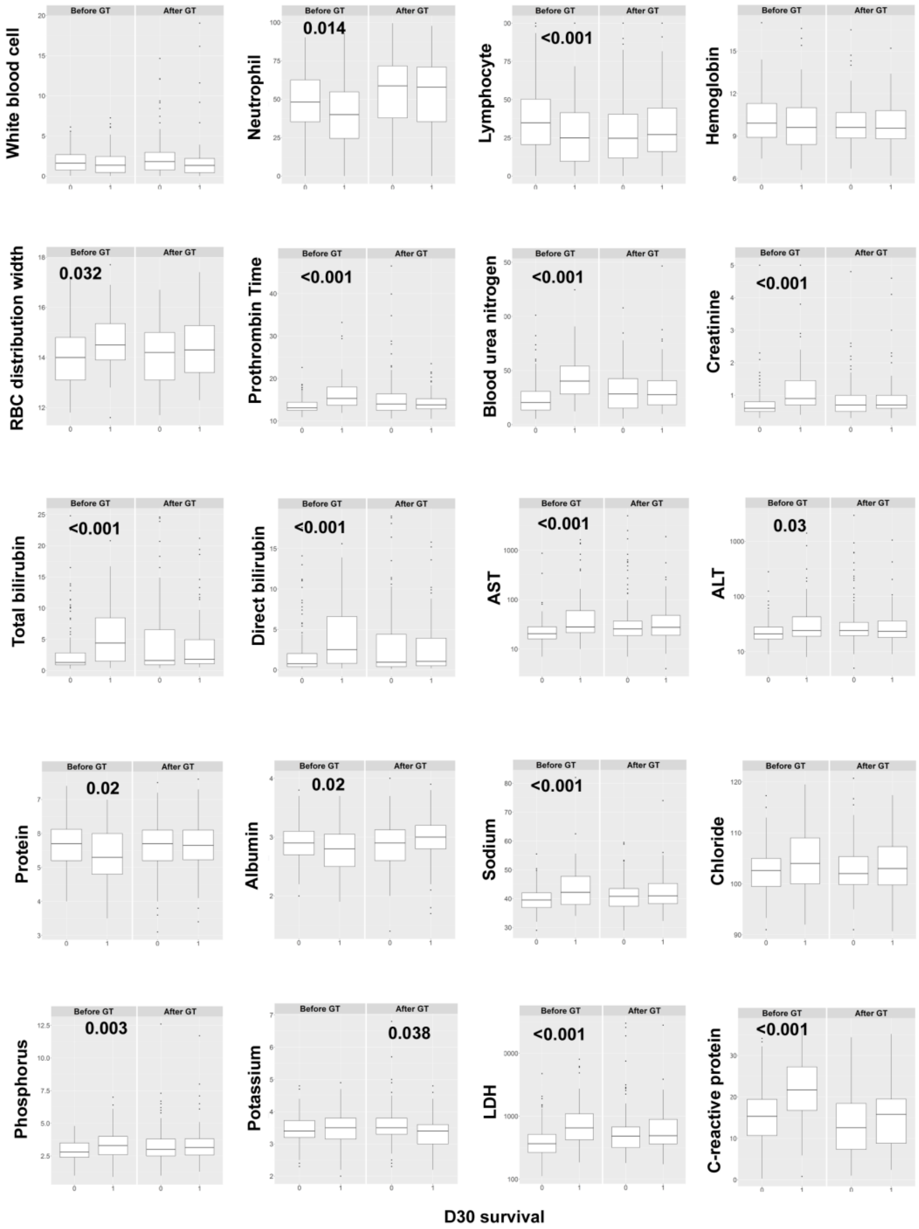

Supplement: S1 File — (PDF) [file pone.0273827.s001.pdf]
